# Generating endogenous *Myh11*-driven Cre mice for sex-independent gene deletion in smooth muscle cells

**DOI:** 10.1172/jci.insight.171661

**Published:** 2023-07-24

**Authors:** Yang Zhao, Guizhen Zhao, Ziyi Chang, Tianqing Zhu, Ying Zhao, Haocheng Lu, Chao Xue, Thomas L. Saunders, Yanhong Guo, Lin Chang, Y. Eugene Chen, Jifeng Zhang

**Affiliations:** 1 Department of Internal Medicine, Cardiovascular Center, University of Michigan Medical Center, Ann Arbor, Michigan, USA.; 2 Department of Pharmacology and; 3 Transgenic Animal Model Core, University of Michigan Medical School, Ann Arbor, Michigan, USA.

**Keywords:** Angiogenesis, Vascular Biology, Cardiovascular disease

## Abstract

Specific and efficient smooth muscle cell–targeted (SMC-targeted) gene deletion is typically achieved by pairing SMMHC-CreER^T2^-Tg mice with mice carrying the loxP-flanked gene. However, the transgene, CreER^T2^, is not controlled by the endogenous *Myh11* gene promoter, and the codon-modified iCreER^T2^ exhibits significant tamoxifen-independent leakage. Furthermore, because the Cre-bearing bacterial artificial chromosome (BAC) is inserted onto the Y chromosome, the SMMHC-CreER^T2^-Tg mice strain can only exhibit gene deletions in male mice. Additionally, there is a lack of *Myh11*-driven constitutive Cre mice when tamoxifen usage is a concern. We used CRISPR/Cas9-mediated homologous recombination between a donor vector carrying the CreNLS^P2A^ or CreER^T2–P2A^ sequence and homologous arm surrounding the translation start site of the *Myh11* gene to generate Cre-knockin mice. The P2A sequence enables the simultaneous translation of Cre and endogenous proteins. Using reporter mice, we assessed Cre-mediated recombination efficiency, specificity, tamoxifen-dependent controllability, and functionality in both sexes. Both constitutive (*Myh11*-CreNLS^P2A^) and inducible (*Myh11*-CreER^T2–P2A^) Cre mice demonstrated efficient, SMC-specific, sex-independent Cre recombinase activity without confounding endogenous gene expression. Combined with recently generated BAC transgenic *Myh11*-CreER^T2^-RAD mice and the *Itga8*-CreER^T2^ mouse models, our models will help expand the research toolbox, facilitating unbiased and comprehensive research in SMCs and SMC-dependent cardiovascular diseases.

## Introduction

Smooth muscle cells (SMCs) are a subtype of spindle-shaped muscle cells enriched in hollow-structured organs such as blood vessels, the bladder, the intestine, and the stomach (1). These are where SMCs own a wide distribution and are responsible for sustaining critical physiological functions, consequently displaying remarkable adaptability in disease circumstances, including cardiovascular diseases (CVDs) (1–3). In the cardiovascular system, SMCs are present in the walls of the aorta and small arteries, regulating blood flow and blood pressure. SMCs also undergo phenotypic switching when exposed to risk conditions such as hypertension, hypercholesterolemia, and smoking, defined by a shift in gene expression patterns and intracellular activities (4–8). This leads to the loss of their contractility and the development of a synthetic phenotype characterized by enhanced proliferation, migration, and matrix formation, contributing to the progression of pathological conditions, including neointima formation and atherosclerosis (3, 9).

To study these complex behaviors of SMCs in greater detail and to understand their role in disease conditions, advanced genetic techniques like the bacteriophage Cre-LoxP system are essential. The bacteriophage Cre-LoxP system is a commonly employed genetic technique for precisely manipulating gene expression in various cell types and developmental stages, providing fresh insights into the role of particular genes in a cell type–dependent manner (10–12). Over the past 2 decades, our lab and others have contributed more than 20 transgenic Cre mouse models driven by SMC-specific promoters to study SMC genes and their contributions to CVDs, to acquire a deeper understanding of the disease-relevant and functional genes involved in SMCs (13–22). Targeted deletion of the gene of interest can be generally achieved by pairing the Cre-bearing mice with mice carrying the loxP-flanked gene. Moreover, by fusing Cre with a mutated (T2) estrogen receptor (ER^T2^), it is possible to remove the gene of interest in a manner that can be manipulated temporally and spatially (23–26). Such controllable expression is precious for SMCs, as they play crucial roles during embryonic development by contributing to the formation and remodeling of various tissues.

The *Myh11* gene, which encodes the smooth muscle myosin heavy chain (SMMHC), serves as an established and reliable biomarker for the maturely differentiated SMCs within both vascular and visceral tissues (15). Although the *Myh11* promoter was also shown to drive Cre activity within lung pericytes, it generally provides an exceptional level of specificity paired with robust expression capabilities that facilitate targeted Cre expression within SMCs (27, 28). This degree of precision and potency surpasses the utility of previously employed *Acta2* (smooth muscle α-2 actin) or *Tagln* (*SM22*α, Transgelin)-driven Cre models. These models, while effective, exhibit broad expression in non-SMC cells, such as myeloid cells and fibroblasts, including myofibroblasts (27). Furthermore, the *Tagln* gene illustrates a higher expression level within embryonic cardiac myocytes than SMCs, thereby constraining its effectiveness in CVD studies (29). To date, the *Myh11*-driven SMMHC-CreER^T2^-Tg transgenic mice (The Jackson Laboratory, strain 019079) are esteemed as the most efficacious models, providing comprehensive coverage of the SMC spectrum while ensuring optimal efficiency and specificity (14).

This mouse model has been extensively used in studies investigating SMC-specific gene activities in CVDs, including studies conducted by our lab (8, 30–32). However, the model has its limitations; the transgene cannot be passed on to females, since it is inserted on the Y chromosome (14, 33). This is particularly important, since CVDs are the leading cause of death worldwide among both males and females (34), and there are well-established sexual disparities in CVD incidence, progression, and outcomes (35–37). Moreover, the sensitivity of SMCs to numerous CVD risk factors, such as hypertension, hypercholesterolemia, and smoking, varies by sex (34, 38). The pathophysiological discrepancies between male and female patients and the absence of sex-oriented CVD research would underestimate the risk in female patients, leaving females likely more vulnerable to improper treatment (39).

Herein, we developed 2 *Myh11*-driven Cre mouse models, including a constitutive *Myh11*-CreNLS^P2A^ KI model and an inducible *Myh11*-CreER^T2–P2A^ KI model, by CRISPR/Cas9-mediated double break and homologous recombination between the donor vector containing the codon-optimized CreNLS^P2A^ or CreER^T2–P2A^ sequence and the homologous arm flanking the ATG translation start site at exon 2 of the endogenous *Myh11* gene. In addition, adding the porcine teschovirus-1 2A (P2A) self-cleaving peptide ensures the production of a bicistronic mRNA transcript that will translate both Cre and intact Myosin-11 proteins (40, 41). The endogenous *Myh11*-driven Cre provides a more physiologically relevant model since it ensures that the Cre expression pattern accurately reflects the endogenous gene expression, preserving tissue-specific and developmental regulation. In contrast, BAC transgenes can sometimes result in ectopic or variable expression patterns due to their large size, potential positional effects, and risks of unintended disruptions to the host genome (42, 43). At the same time, the model bypasses the limitation of the SMMHC-CreER^T2^ BAC transgene being inserted on the Y chromosome, allowing for sex-independent, SMC-specific Cre recombinase activity. This is particularly important for studying sex-specific differences in SMC biology and related CVDs. Importantly, the constitutive *Myh11*-CreNLS^P2A^ KI model provides a valuable tool for studying SMC gene function where the high dose of tamoxifen (TMX, 50 to 75 mg/kg/day) usage is a concern. On the other hand, the inducible *Myh11*-CreER^T2–P2A^ KI model offers temporal control over gene expression, accommodating studies that require precise timing. These new models will likely help promote more impartial, systematic, and precise preclinical studies in SMCs and SMC-related CVDs.

## Results

### Generation and characterization of Myh11-CreNLS^P2A^–KI mice.

**Figure 1:**
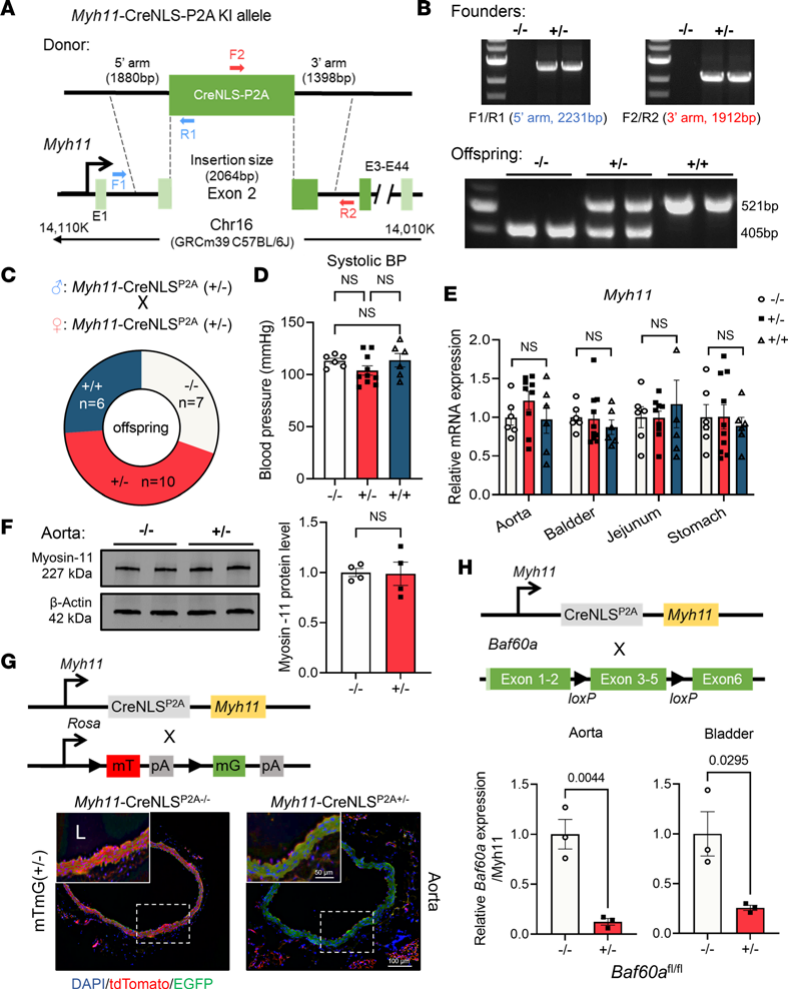
Generation and characterization of *Myh11*-CreNLS^P2A^ KI mice. (**A**) Schematic illustration of the strategy for generating endogenous *Myh11*-driven smooth muscle–targeted *Myh11*-CreNLS^P2A^ KI mice. (**B**) PCR confirmation of the *Myh11*-CreNLS^P2A^ KI insertion site for F0 founder mice and identification of *Myh11*-CreNLS^P2A–/–^, *Myh11*-CreNLS^P2A+/–^, and *Myh11*-CreNLS^P2A+/+^ offspring. (**C**–**F**) Effect of *Myh11*-CreNLS^P2A^ KI on offspring Mendelian distribution (**C**), systolic blood pressure (**D**), endogenous *Myh11* gene expression in the aorta, bladder, jejunum, and stomach (**E**) (*n* = 6–10 per group), and Western blot quantification of Myosin-11 (**F**) (coded by *Myh11*) protein abundance in the aorta (*n* = 4 per group). (**G**) *Myh11*-CreNLS^P2A^ KI mice were crossbred with ROSA26-driven mT/mG reporter mice. Cre activity in the aorta is proportional to the loss of tdTomato (red) signal and gain of EGFP (green) signal. Scale bars: 50 μm (upper left); 100 μm (lower right). L, aortic lumen. (**H**) *Myh11*-CreNLS^P2A^ KI mice were crossbred with BAF60a-floxed mice, followed by quantification of KO efficiency in the aorta and bladder using qPCR (*n* = 3 per group). Data are presented as mean ± SEM. Unpaired, 2-tailed Student’s *t* test (**F** and **H**), 1-way ANOVA (**D**), and 2-way ANOVA (**E**) followed by the Tukey test were used.

We generated the *Myh11*-CreNLS^P2A^–KI mice by microinjecting a donor assembly consisting of sgRNA, and Cas9 protein (RNP, ribonucleoprotein) into fertilized mouse eggs (Supplemental Figure 1, A and B; supplemental material available online with this article; https://doi.org/10.1172/jci.insight.171661DS1). The Cre transgene was strategically inserted into the endogenous *Myh11* gene immediately after the first codon ATG in exon 2 (Figure 1A). Identification of both the founder and offspring was achieved through genotyping PCR and sequencing (Figure 1B). Heterozygous male founders with a C57BL/6J × SJL background were backcrossed to C57BL/6J females across multiple generations before determining the efficiency and specificity of Cre-mediated reporter gene expression. No off-target cleavage was detected at the predicted off-target sites on chromosome 16 and exons in other chromosomes in all F1 generations, as confirmed by PCR and sequencing (Supplemental Figure 1, A and B).

Heterozygote (*Myh11*-CreNLS^P2A^^+/–^) and homozygote (*Myh11*-CreNLS^P2A^^+/+^) offspring were viable (Figure 1C), with smooth muscle functions largely preserved. For example, systolic blood pressure remained consistent across WT (*Myh11*-CreNLS^P2A^^–/–^), heterozygous, and homozygous mice (Figure 1D). Furthermore, the insertion of *Myh11*-CreNLS^P2A^ had a minimal impact on endogenous *Myh11* mRNA and Myosin-11 protein levels in SMC-enriched tissues, such as the aorta and intestine (Figure 1, E and F).

Subsequently, *Myh11*-CreNLS^P2A^ KI mice were crossbred with mT/mG, a double-fluorescent Cre reporter mouse that expresses membrane-targeted Tomato (mT) prior to Cre-mediated excision, and membrane-targeted green fluorescent protein (mG) after excision (44). Cre activity was observed in the medial layer of the aorta, where SMCs are located (Figure 1G). To quantify Cre efficiency, *Myh11*-CreNLS^P2A^ KI mice were crossbred with *Baf60a*-floxed mice. qPCR revealed significant reductions in *Baf60a* expression in the aorta (by 87.67% ± 15.16%, *P* = 0.0044) and bladder (by 74.33% ± 22.42%, *P* = 0.0295; Figure 1H), underlining the high efficiency and SMC-specificity of the Cre recombinase.

### Comparative analysis of Cre specificity and efficiency between Myh11-CreNLS^P2A^ KI mice and SMMHC-CreER^T2^-Tg mice.

**Figure 2:**
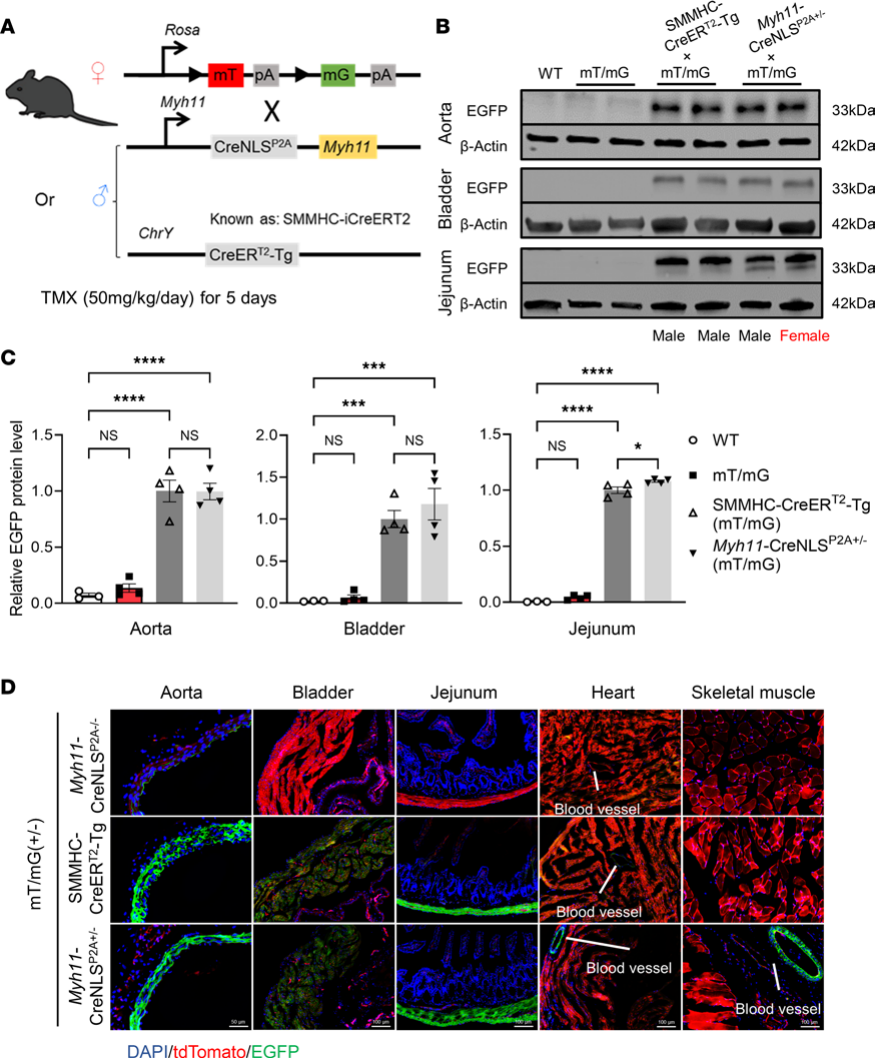
Comparative analysis of Cre-dependent specificity and efficiency between *Myh11*-CreNLS^P2A^ KI mice and SMMHC-CreER^T2^-Tg mice. (**A**) *Myh11*-CreNLS^P2A^ KI and SMMHC-CreER^T2^-Tg mice were crossbred with mT/mG reporter mice. Mice were i.p. administered 50 mg/kg/day tamoxifen/corn oil for 5 consecutive days, followed by a 1 week of rest, frozen sectioning, and protein isolation from various tissues. (**B**) Western blot analysis of EGFP and β-actin in the aorta, bladder, and jejunum. (**C**) Quantification (*n* = 3–4 per group) of **B**. (**D**) tdTomato and EGFP signal in frozen sections of the aorta, bladder, jejunum, heart, and lower limb isolated from *Myh11*-CreNLS^P2A^ KI and SMMHC-CreER^T2^-Tg mice, respectively, crossbred with mT/mG reporter mice. Data are presented as mean ± SEM. One-way ANOVA followed by the Tukey test (**C**). ***P < 0.001; ****P < 0.0001.

The SMMHC-CreER^T2^-Tg mouse model is widely recognized as possessing the highest SMC specificity and efficiency for targeted gene deletion (14). For a comprehensive comparison, we crossbred both the *Myh11*-CreNLS^P2A^ KI mice and SMMHC-CreER^T2^-Tg with mT/mG reporter mice, subsequently administering TMX (50 mg/kg/day for 5 days, i.p.; Figure 2A) (13). Western blot analysis, examining EGFP and β-actin, revealed remarkably specific and pronounced Cre recombinase activity in the aorta, bladder, and gastrointestinal (GI) tract (Figure 2, B and C). Notable, the *Myh11*-CreNLS^P2A^ KI mice displayed comparable EGFP expression between male and female mice (Figure 2B). Furthermore, examination of frozen sections from *Myh11*-CreNLS^P2A^ KI mice crossbred with mT/mG reporter mice revealed a comparable increase in EGFP fluorescence and decreases in tdTomato fluorescence in the SMC segment of the aorta, bladder, and jejunum, akin to that observed in the SMMHC-CreER^T2^-Tg mice. Importantly, Cre recombinase activity was SMC-specific and was conspicuously absent in cardiomyocytes and skeletal muscles (Figure 2D).

### Generation and characterization of Myh11-CreER^T2–P2A^ KI mice.

Targeting the same insertion site by Cas9/sgRNA as described earlier, we successfully generated TMX-inducible *Myh11*-CreER^T2–P2A^ KI mice (Supplemental Figure 1, A and C, and Supplemental Figure 3A). Similarly, off-target indel events were not detected at the predicted off-target sites (Supplemental Figure 2B). We verified that the insertion was at the correct site and confirmed the presence of WT (*Myh11*-CreER^T2–P2A^^–/–^), heterozygous (*Myh11*-CreER^T2–P2A^^+/–^), and homozygous (*Myh11*-CreER^T2–P2A^^+/+^) offspring (Supplemental Figure 3B). Moreover, there were no noticeable variations in *Myh11* expression or systolic blood pressure between WT and heterozygotes *Myh11*-CreER^T2–P2A^ KI mice (Supplemental Figure 3, C–F).

**Figure 3:**
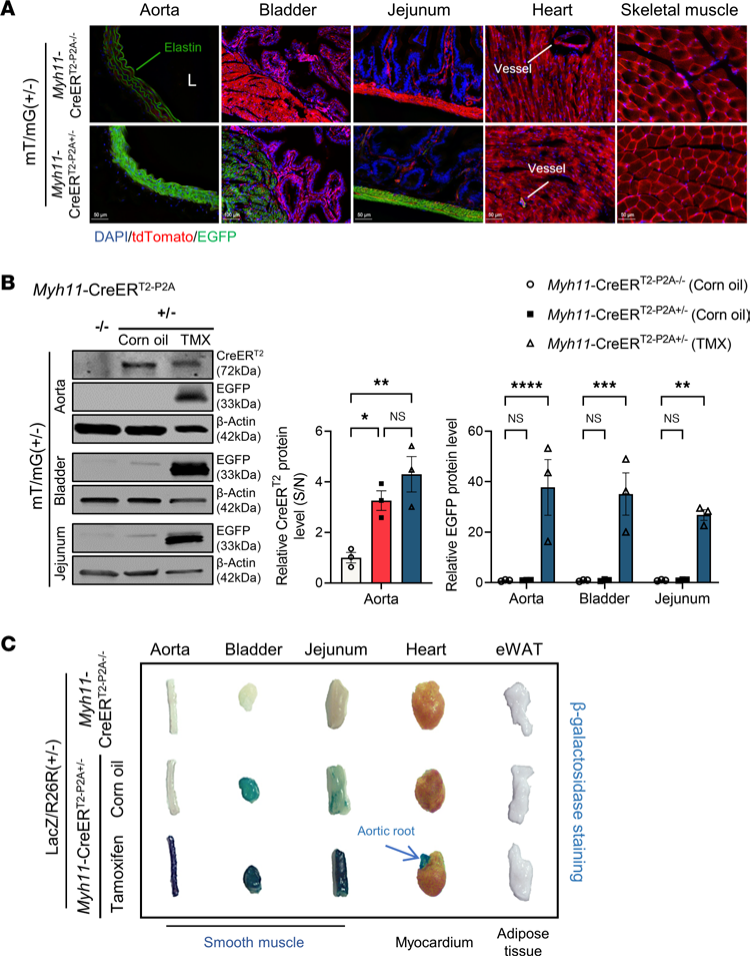
Characterization of tamoxifen-inducible Cre activity in *Myh11*-CreER^T2–P2A^ KI mice. *Myh11*-CreER^T2–P2A^ KI mice were crossbred with mT/mG or LacZ reporter mice. Mice were i.p. administered 50 mg/kg/day tamoxifen/corn oil for 5 consecutive days, followed by1 week of rest, X-gal staining, frozen sectioning, or protein isolation from different tissues. (**A**) tdTomato and EGFP signal in frozen sections of aorta, bladder, jejunum, heart, and lower limb isolated from *Myh11*-CreER^T2–P2A+/–^ or *Myh11*-CreER^T2–P2A–/–^ mice crossbred with mT/mG reporter mice. Scale bars: 50 μm (aorta, jejunum, heart, skeletal muscle); 100 μm (bladder). L, aortic lumen. (**B**) Western blot analysis of CreER^T2^ or EGFP and β-actin in the aorta, bladder, and jejunum, followed by quantification (*n* = 3 per group). *P < 0.05; **P < 0.01; ***P < 0.001; ****P < 0.0001. (**C**) X-gal (β-galactosidase) staining to visualize Cre recombinase activity in the aorta, bladder, jejunum, heart, and eWAT from *Myh11*-CreER^T2–P2A+/–^ or *Myh11*-CreER^T2–P2A–/–^ mice injected with corn oil or tamoxifen. Data are presented as mean ± SEM. One-way ANOVA for CreER^T2^ and 2-way ANOVA for EGFP quantification in **B**, followed by the Tukey test.

*Myh11*-CreER^T2–P2A^^+/–^ mice were then crossbred with both mT/mG and LacZ reporter mice (R26R; Supplemental Figure 3G), followed by TMX administration (50 mg/kg/day for 5 days, i.p.). Frozen sections from *Myh11*-CreER^T2–P2A^^+/–^ and heterozygous mT/mG reporter mice displayed smooth muscle–specific EGFP expression and loss of tdTomato signal in the aorta, bladder, and jejunum but not in cardiomyocytes or skeletal muscles (Figure 3A).

In addition, Western blot analysis of EGFP and β-actin from *Myh11*-CreER^T2–P2A^ KI mice crossbred with mT/mG reporter mice indicated robust TMX-inducible Cre recombinase activity in the aorta, bladder, and GI tract (Figure 3B). In agreement prior reports (16, 22), we noticed mild endogenous Cre activity in the aorta, bladder, and GI tract of non-TMX-treated *Myh11*-CreER^T2–P2A^ mice, likely due to TMX-independent CreER^T2^ nuclear translocation activity (Figure 3B). We assessed both endogenous and inducible Cre recombinase activity at the organ level using β-galactosidase staining (Figure 3C). Our analysis confirmed high TMX–induced Cre activity in SMC-rich organs and negligible leakage into the myocardium or epididymal white adipose tissue (eWAT). This was supported by an increase in the blue signal, relative to the baseline LacZ/R26R (+/–) Cre-negative control. However, consistent with earlier reports (45), we noted TMX-independent CreER^T2^ leakage in the bladder and GI tract (Figure 3C). This finding highlights the need for meticulous care when utilizing the *Myh11*-CreER^T2–P2A^ KI mice in studying genes implicated in important visceral SMC functionality.

## Discussion

In this study, we generated 2 endogenous *Myh11* promoter–driven Cre mouse models. One of these models exhibits constitutive Cre activity (*Myh11*-CreNLS^P2A^ KI mouse), and the other shows TMX-induced Cre activation (*Myh11*-CreER^T2–P2A^ KI mouse). Our results demonstrate that both models exhibited high efficiency, specificity, and sex-independent Cre recombinase activity in targeting SMCs, comparable with the widely used SMMHC-CreER^T2^-Tg mice. The generation of these mouse models is of particular importance, given the vastly underexplored role of SMCs in CVDs, especially in females. The ability to precisely and inducibly manipulate gene expression in SMCs by *Myh11*-CreER^T2–P2A^ KI mice will facilitate a more comprehensive understanding of the molecular mechanisms underlying dynamic SMC changes. The 2 *Myh11*-Cre mouse lines present valuable resources to explore the role of SMCs in the pathogenesis of various diseases, including cardiovascular, respiratory, GI-related, and many others. The sex-independent nature of these mouse models also allows the examination of sex-specific mechanisms in disease progression, providing insights into the dynamic pathological changes in SMCs and aiding the identification of potential therapeutic targets. Meanwhile, the constitutive *Myh11*-CreNLS^P2A^ KI we developed addresses some of the issues related to the usage of TMX, a commonly used agent for inducing Cre recombinase activity. Notably, a high dose of TMX has been reported to exhibit sex-dependent differences and off-targets potentially influencing cell proliferation, apoptosis, and angiogenesis, which could confound the interpretation of results in studies related to CVDs (46–48). The model helps to minimize potential confounding factors, enhancing the reliability and clarity of experimental outcomes.

In the past 2 decades, efforts to identify reliable SMC-specific Cre and CreER^T2^ mouse models have utilized 3 major promoters: *Tagln*, *Acta2*, and *Myh11* (27). It is recognized by the field that the Cre driven by *Myh11* promoter shows both reasonable SMC specificity and efficiency, and thus, the SMMHC-CreER^T2^-Tg KI mouse has been widely used for SMC-specific gene deletion (14, 27). Although the SMMHC-CreER^T2^-Tg model is advantageous in some respects, it has 2 major limitations. Firstly, it introduces a substantial fragment insertion into the Y-chromosome, rendering it incompatible with female participants and unsuitable for studies requiring a littermate control. Secondly, due to the large size of these insertions and potential positional effects, the SMMHC-CreER^T2^-Tg model carries the risk of ectopic or variable expression patterns (16, 33, 49). The recent reported transgenic *Myh11*-CreER^T2^-RAD mice generated by G. Owens’ lab resolved the first limitation by inserting the BAC construct into chromosome 2 (22). In addition, J. Miano’s lab reported a potentially new *Itga8*-CreER^T2^ mouse in which the CreER^T2^ is driven by endogenous *Itga8* promoter and shows vascular SMC–preferential (VSMC-preferential) Cre activity, enabling the study of previously unexplorable critical SMC genes such as serum response factor (SRF) due to the lethal GI phenotype (16). However, given that the *Itga8*-CreER^T2^ sequence is followed by a poly A signal, the endogenous *Itga8* was knocked out by the insertion. Caution will be needed to differentiate the effect of potential *Itga8* downregulation when used for SMC-specific gene deletion, especially under disease conditions.

While our constitutive *Myh11*-CreNLS^P2A^ KI model and inducible *Myh11*-CreER^T2–P2A^ KI model present considerable advantages, we acknowledge the inherent complexities and variations in biological research that necessitate the customization of Cre driver mouse models to cater to specific experimental settings. The constitutive model offers a robust, durable platform devoid of inducer-associated side effects and attributable primarily to the codon-improved Cre recombinase (iCre) and the introduction of the nuclear localization peptide (NLP) sequence, showing enhanced efficiency of Cre-mediated recombination. On the other hand, the inducible *Myh11*-CreER^T2–P2A^ KI model provides a degree of temporal control, enabling targeted investigation into the functional dynamics of SMC genes across developmental stages. Furthermore, this model mitigates potential interference from compensatory mechanisms from chronic gene KO and facilitates the exploration of genes integral to embryonic development. Manipulation of such genes, if done so through a constitutive Cre model, may lead to embryonic lethality. In addition, the *Myh11*-CreER^T2–P2A^ KI model does demonstrate a certain degree of TMX-independent leaky activity in SMCs, as seen in the SMMHC-CreER^T2^-Tg and *Myh11*-CreER^T2^-RAD mice, and, like most SMC Cre drivers, they also display higher activity in visceral SMC (50). This characteristic requires careful consideration, especially in studies focusing on disorders where visceral SMC may play a pivotal role in pathology. For these specific scenarios, the *Itga*8-Cre mouse model could provide a more suitable alternative due to its vascular preference and lower activity in visceral SMCs.

Despite the established roles of SMCs in embryogenesis and organ development, new models of *Myh11*-driven Cre, including those developed by our team, have not yet been explored within these contexts. Utilizing these models for developmental studies could offer profound insights, particularly when it comes to lineage tracing of SMCs in developmental disorders. However, a prudent approach would be to thoroughly evaluate these models during developmental stages prior to conducting related experiments. With this in mind, we propose that our models could serve as potent investigative tools for future SMC lineage tracing during development. The use of endogenous *Myh11*-driven Cre in our models presents a potential advantage, as it likely provides a more physiologically relevant representation of these processes. It assures that the Cre expression pattern accurately mirrors endogenous gene expression, thereby maintaining tissue-specific and developmental regulation. This feature can be instrumental in ensuring a faithful reflection of in vivo conditions, thereby improving the reliability and relevance of experimental outcomes derived from these models. However, it is important to note that the incorporation of the P2A sequence between *Myh11* and CreER^T2^ might affect *Myh11* gene expression or result in incomplete protein cleavage under pathophysiological conditions, which could subsequently impact SMC functionality (41). Comprehensive evaluations under diverse disease scenarios in future studies are anticipated to unlock the full potential of the new Cre models.

In summary, the potentially novel *Myh11*-CreNLS^P2A^ and *Myh11*-CreER^T2–P2A^ mouse models provide powerful research tools for investigating SMC biology and related cardiovascular disorders. Coupled with the recently developed *Myh11*-CreER^T2^-RAD and the VSMC-specific *Itga8*-CreER*T2* models, these resources enrich and enhance the repertoire of transgenic animal models available for SMC and SMC-linked CVD research. Future investigations employing these models are expected to illuminate intricate molecular mechanisms that govern both the physiological functionality and pathological dysfunction of SMCs, thereby propelling our comprehension of these multifaceted diseases to new heights.

## Methods

Supplemental Methods are available online with this article.

### Antibodies and reagents.

Rabbit IgG (catalog 2729), GFP (D5.1) rabbit mAb (catalog 2956), and mouse β-actin mAb (catalog 3700) were obtained from Cell Signaling Technology (CST). SMMHC rabbit pAb (catalog 21404-1-AP) and Cre recombinase pAb (catalog PA5-32245) were sourced from Thermo Fisher Scientific. TMX (catalog 13258) was purchased from Cayman.

### Animal experiments.

The F0 founders of *Myh11*-CreNLS^P2A^ and *Myh11*-CreER^T2–P2A^ KI mice were generated by the University of Michigan Transgenic Animal Model Core by microinjection CRISPR components and donor vector into fertilized mouse eggs of C57BL/6J mouse. Male founders of *Myh11*-CreNLS^P2A^ and *Myh11*-CreER^T2–P2A^ KI mice were bred with female C57BL/6J mice (strain 000664, The Jackson Laboratory) over multiple generations. Offspring were genotyped and used for downstream characterization or crossbreeding with the corresponding reporter mice. mT/mG (strain 007676), LacZ (strain 003474), and control SMMHC-CreER^T2^-Tg (strain 019079) mice were purchased from The Jackson Laboratory. *Baf60a* floxed (*Baf60a*^fl/fl^) mice with a C57BL/6J background were obtained as previously described and are also available from The Jackson Laboratory (strain 036171) (51). TMX (50 mg/kg/day) or corn oil was injected i.p. for 5 consecutive days, followed by a 1-week resting period to induce CreER^T2^ nuclear translocation (30). Systolic blood pressure was measured using the BP-2000 Blood Pressure Analysis System from Visitech Systems Inc. Mice were maintained under a regular 12-hour light/12-hour dark cycle at 22°C within a pathogen-free environment.

### Histological analysis.

After euthanasia, mice were perfused with ice-cold saline via the left ventricle. The aorta, bladder, heart, jejunum, limbs, and adipose tissue were excised, formalin-fixed, dehydrated in gradient sucrose PBS solution (15% and 30%), and then embedded in paraffin or OCT (Thermo Fisher Scientific, 14-373-65) before sectioning. Sections were rehydrated in PBS for 10 minutes. tdTomato and EGFP signals were imaged using a Keyence BZ800 fluorescence microscope.

### Protein extraction and Western blotting.

Tissues were lysed in RIPA buffer (Thermo Fisher Scientific, 89901) supplemented with a protease inhibitor (Roche, 11873580001). Protein extracts were run on SDS-PAGE gels and subsequently transferred to nitrocellulose membranes. The membranes were blocked with 5% milk or BSA for 1 hour at room temperature, followed by incubation with primary antibody overnight at 4°C, and then they were incubated with fluorescence-labeled secondary antibodies (Li-Cor Bioscience, 1:5,000 dilution) for 1–2 hours at room temperature. Membranes were scanned using the LI-COR DLx Odyssey imaging system. Quantification was carried out using LI-COR Empiria Studio.

### mRNA isolation and qPCR analysis.

Total RNA from tissues was extracted using TRIzol (Invitrogen, 15596018) and the RNeasy Mini Kit (Qiagen, 74106). mRNA was reverse transcribed with the SuperScript III kit (Thermo Fisher Scientific, 18080051) using random hexamers. Measurement of cDNA abundance was performed in a Bio-Rad Realtime PCR Detection System, using SYBR Green Fast qPCR Mix (Abclonal, RK21203). qPCR primers are outlined in Supplemental Table 1.

### Tissue isolation and X-gal staining.

After euthanization, mice were perfused with ice-cold saline via the left ventricle. Next, the aorta, heart, bladder, jejunum, limbs, and adipose tissues were dissected and fixed in formalin for 5 minutes, followed by three 5-minute PBS washes. Tissues were incubated in 5-bromo-4-chloro-3-indolyl β-d-galactopyranoside–containing (X-gal–containing) solution in a 37°C water bath for 30–60 minutes, with duration dependent on tissue type. The X-gal–containing solution was prepared according to the manufacturer’s protocol (Thermo Fisher Scientific, K146501).

### Statistics.

Statistical analyses were performed using GraphPad Prism 9 software. Data were examined for normality with the Shapiro-Wilk test and were compared against “normality versus lognormality” when data were normalized to control values. Outliers were detected using the ROUT method at a *q* value of 1% for raw data. For comparison between 2 means, an unpaired 2-tailed Student’s *t* test was employed. One-way ANOVA was utilized to compare means across multiple groups, while nonlinear regression or 2-way ANOVA were used for data featuring 2 independent variables. To compare individual means, post hoc Tukey tests were applied. Continuous variables were expressed as mean ± SEM. A *P* value less than 0.05 was considered statistically significant. For in vitro results accompanied by statistical representation, at least 3 independent replicates were performed.

### Study approval.

All animal procedures were performed following the protocols approved by IACUC at the University of Michigan.

### Data availability.

Values for all data points found in graphs can be found in the supporting data values file and will be available from the corresponding author upon request.

## Author contributions

Yang Zhao, GZ, ZC, and JZ conducted the experiments and performed data analysis; Yang Zhao and JZ prepared the manuscript; TZ, Ying Zhao, HL, CX, TLS, YG, and LC provided experimental and technical support; YEC and JZ edited the manuscript; YEC and JZ contributed to the experimental design.

## Supplementary Material

Supplemental data

Supporting data values

## Version 1 06/08/2023

In-Press Preview

## Version 2 07/24/2023

Electronic publication
