# Supplementary material for: Generating endogenous Myh11-driven Cre mice for sex-independent gene deletion in smooth muscle cells
Source: JCI Insight. 2023 Jul 24;8(14):e171661. doi: 10.1172/jci.insight.171661 (PMC10443793; doi:10.1172/jci.insight.171661)
Supplement: Supplemental data [file jciinsight-8-171661-s089.pdf]

Supplemental materials

Generating endogenous *Myh11*-driven Cre Mice for Sex-Independent Gene Deletion in Smooth Muscle Cells

Yang Zhao[1,2] †, Guizhen Zhao[1] †, Ziyi Chang[1] †, Tianqing Zhu[1], Ying Zhao[1], Haocheng Lu[1], Chao Xue[1], Thomas L. Saunders[3], Yanhong Guo[1], Lin Chang[1], Y. Eugene Chen[1] *, Jifeng Zhang[1] *

[1]Department of Internal Medicine, Cardiovascular Center, University of Michigan Medical Center, Ann Arbor, MI, USA.

[2]Department of Pharmacology, University of Michigan Medical School, Ann Arbor, MI, USA.

[3]Transgenic Animal Model Core, University of Michigan Medical School, Ann Arbor, MI, USA.

†These authors contributed equally to this work.

*These authors share senior authorship.

*** Correspondence:**

Y. Eugene Chen, Ph.D., University of Michigan Medical School, 2800 Plymouth Road, Ann Arbor, MI 48109, e-mail: echenum@umich.edu, tel: +1 734-936-9548

Jifeng Zhang, Ph.D., University of Michigan Medical School, 2800 Plymouth Road, Ann Arbor, MI 48109, e-mail: jifengz@umich.edu, tel: +1 734-647-8975

Supplemental Methods

Insertion site and guide RNA design

Sequences upstream and downstream of the ATG start site for *Myh11* exon2 were uploaded to http://crispor.tefor.net/ for guide RNA (gRNA) designing. gRNA was selected among the top candidates based on the criteria that there should be at least three mismatches next to PAM sequences. The same gRNA and insertion site was chosen for generating the *Myh11*-CreNLS^{P2A} and *Myh11*-CreER$^{T2\text{-}P2A}$ mice.

Input sequence:

TTCACTCCACAGGGGACCACCAGACATCATGGCGCAGAAAGGGCAGCTCAGCGATGATGA

sgRNA:

CAGGGGACCACCAGACATCA TGG (PAM)

Off-targets for 0-1-2-3-4 mismatches (+ next to PAM):

0 - 0 - 1 - 15 - 279 (0 - 0 - 0 - 1 - 3)

CRISPR/Cas9 off-target validation

Genomic DNA from the *Myh11*-CreNLS^{P2A} and *Myh11*-CreER$^{T2\text{-}P2A}$ F1 offspring was extracted and quality-checked via spectrophotometry and gel electrophoresis. Off-target predictions and primer designs for possible off-target sites, particularly on chromosome 16 and other exon-related regions, were performed using the CRISPOR tool (http://crispor.tefor.net/). Primers were used to amplify potential off-target regions. Amplified products were evaluated by size using agarose gel electrophoresis, with no anomalous sizes detected. Gel-purified PCR products were sequenced with Sanger sequencing, and the data was further compared to reference genomes to identify off-target modifications.

Supplemental Figures and Tables

Supplemental Figures

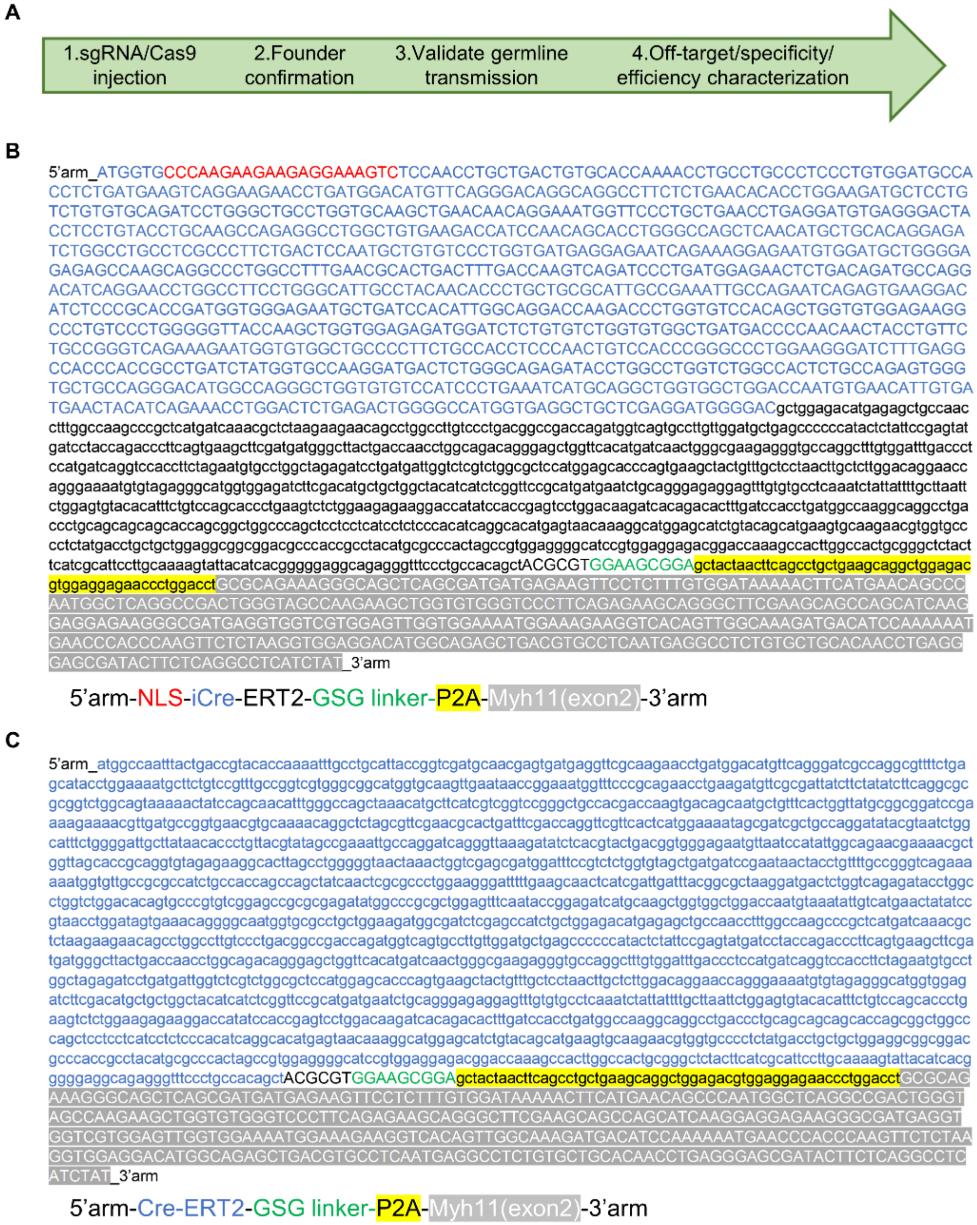

Supplemental Figure 1. Strategy to generate the *Myh11*-CreNLS^{P2A} KI and *Myh11*-CreER$^{T2\text{-}P2A}$ KI mice. (A) Schematic illustration of the pipeline for generating and characterizing the *Myh11*-CreNLS^{P2A} and *Myh11*-CreER$^{T2\text{-}P2A}$ KI mice. (B) Insertion sequence for *Myh11*-CreNLS^{P2A} KI mice. (C) Insertion sequence for *Myh11*-CreER$^{T2\text{-}P2A}$ KI mice.

A

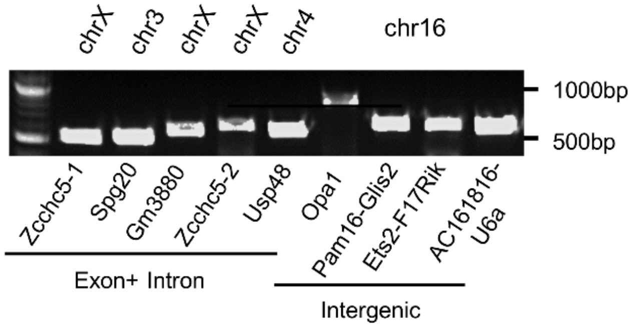

PCR products of predicted exon mutations and intergenic mutations located on Chr16.

B

Primer sequence	Target name	Genome loci (mm9)	CreNLS^{P2A}	CreER^{T2-P2A}
GTACCCTGGTTGTCTGTGGG	Zcchc5_chrX-F	chrX:104034663-104034685:-	None	None
GCAGGCTCAAGTGAAAAGGC	Zcchc5_chrX_R		None	None
TGCGTTTACCTATCCCTGAT	Spg20_chr3_F	chr3:54916584-54916606:-	None	None
CTCAGAACTAGGAGCACTCA	Spg20_chr3_R		None	None
CCCAGTCATTCCTCTAAGGT	Gm3880_chrX_F	chrX:99882005-99882027:-	None	None
GAGTGAGGCCTTAACTCTCT	Gm3880_chrX_R		None	None
GATCTCCTGGGATTCCTTGA	Zcchc5_chrX_F	chrX:104034582-104034604:-	None	None
CCGTTTCCTCAAAAGATGGT	Zcchc5_chrX_R		None	None
AAACCCAGAGTTTGTGGCTTTGTAACA	Usp48_chr4_F	chr4:137213875-137213897:-	None	None
CGGGTAGAGCTGGGGATGTGG	Usp48_chr4_R		None	None
CCCTGGAAGCAATGATGTAC	Pam16-Glis2_c16_F	chr16:4620799-4620821:+	None	None
ATGCTCTATCAGTCTGCCAA	Pam16-Glis2_c16_R		None	None
GGTCTGGCCTAAGGTGCTC	Opa1_chr16_F	chr16:29650111-29650133:+	None	None
TCCAGGCAGGGAAACAAGTC	Opa1_chr16_R		None	None
CTGTACCCCAAACCTCCACC	Ets2-F17Rik_c16_F	chr16:95970651-95970673:-	None	None
TGCCAGCATCTGACTGAAGG	Ets2-F17Rik_c16_F		None	None
GAGGCCACATGGTTAACCTGA	AC161816-U6_chr16_F	chr16:40197051-40197073:+	None	None
TTCAGAGTGCTTGAGCCTGG	AC161816-U6_chr16_R		None	None

Supplemental Figure 2. Off-target characterization of the *Myh11*-CreNLS^{P2A} KI and *Myh11*-CreER^{T2-P2A} KI mice. (A) PCR for potential off-targets on chromosome 16 (exon, intron, intergenic) and exon-related off-targets located on all other chromosomes. (B) Table of PCR primers for detecting potential off-targets generated by Cas9/sgRNA. “None” stands for no indel events identified by sequencing.

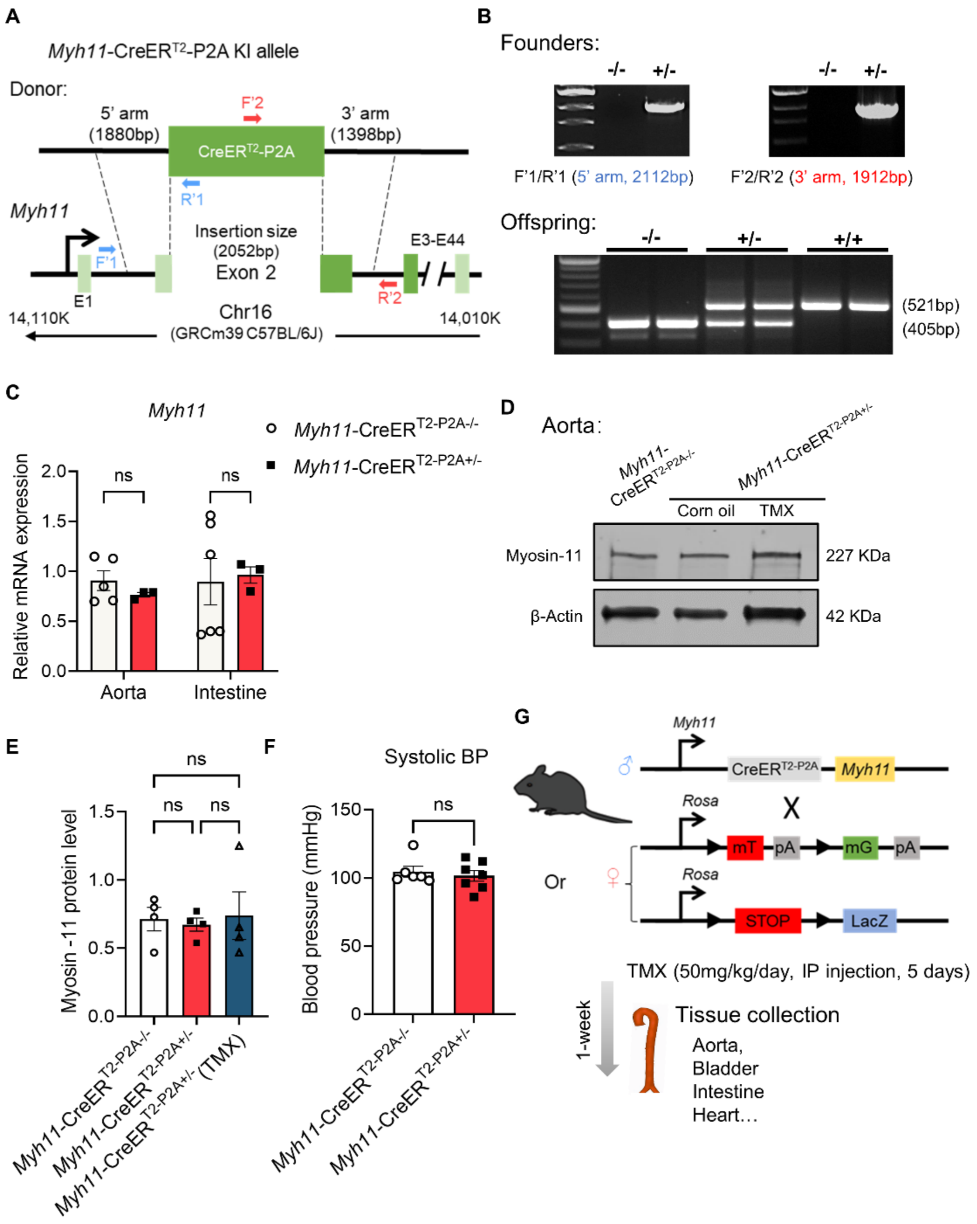

Supplemental Figure 3. Generation and characterization of the *Myh11*-CreER$^{T2\text{-}P2A}$ KI mice. (A) Schematic illustration of the pipeline for generating endogenous *Myh11*-driven, tamoxifen-inducible, smooth muscle-targeted *Myh11*-CreER$^{T2\text{-}P2A}$ KI mice. (B) PCR confirmation of the KI insertion site for F0 founder mice and genotyping of F1 *Myh11*-CreER$^{T2\text{-}P2A-/-}$, *Myh11*-CreER$^{T2\text{-}P2A+/-}$, and *Myh11*-CreER$^{T2\text{-}P2A+/+}$ offsprings. (C) Quantitative real-time PCR of *Myh11* expression in the aorta and small intestine (jejunum) from *Myh11*-CreER$^{T2\text{-}P2A-/-}$ and *Myh11*-CreER$^{T2\text{-}P2A+/-}$ mice (n= 3-6 per group). (D) Western blot of Myosin-11 and β-Actin in the aorta from *Myh11*-CreER$^{T2\text{-}P2A-/-}$ and *Myh11*-CreER$^{T2\text{-}P2A+/-}$ mice, followed by (E) quantification (n= 4 per group). (F) The effect of CreER$^{T2\text{-}P2A}$ knock-in on systolic blood pressure (n= 6-7 per group). (G) Schematic illustration of *Myh11*-CreER$^{T2\text{-}P2A}$ KI mice crossbred with mT/mG or LacZ reporter strains underwent a treatment regimen involving tamoxifen administration (50mg/kg/day) for five consecutive days. After one week of rest, various tissue samples were collected for X-gal staining, frozen sectioning, or protein extraction. Data are presented as mean ± SEM. Unpaired Student's t-test for (F), 1-way ANOVA for (E), and 2-way ANOVA for (C) followed by Tukey's test.

Supplemental Tables

Supplemental Table 1. PCR & qPCR primers

Target	Forward Sequence	Reverse Sequence	Notes
iCreNLS-P2A (5'-F1/R1)	CACCAAAACCTGCCTGCCCTCC	CAGTTGTGGGGTTAGGTGGTGTCA	
iCreNLS-P2A (3'-F2/R2)	CGGGCTCTACTTCATCGCATTCCT	GTTGTGGCCATAGAGCATCCTTTTCA	Shared F&R
CreERT2-P2A (5'-F'1/R'1)	AAAGCACAAAGACCCGAGATCT	GGCAAATTTTGGTGTACGGTCA	
CreERT2-P2A (3'-F'1/R'1)	CGGGCTCTACTTCATCGCATTCCT	GTTGTGGCCATAGAGCATCCTTTTCA	Shared F&R
gt-Cre-WT	GCCTGGGTTTCATTCTGTGTCTTTCAT	GACCACCTCATCGCCCTTCTCCTCCTT	Shared R
gt-Cre-KI	TGGCCCAGCTCCTCCTCATCCTCT	GACCACCTCATCGCCCTTCTCCTCCTT	Shared R
qPCR-*Myh11*	ATCGCCCAGAAAAACAATGCCCTAAA	GCGTATCCTCCAGCTCCGTCTTGA	
qPCR-*Baf60a*	GGCGGTCCAAAATCGAAATC	ACCAGTTCCCGAATCCTTTG	
